# Supplementary material for: Region-specific generation and retention of acetylated cohesin shape the genome-wide pattern of sister chromatid cohesion
Source: Nucleic Acids Res. 2026 Oct 31;54(20):gkag1061. doi: 10.1093/nar/gkag1061 (PMC13722876; doi:10.1093/nar/gkag1061)
Supplement: Supplementary file 1 [file gkag1061_supplemental_file.pdf]

Figure S1

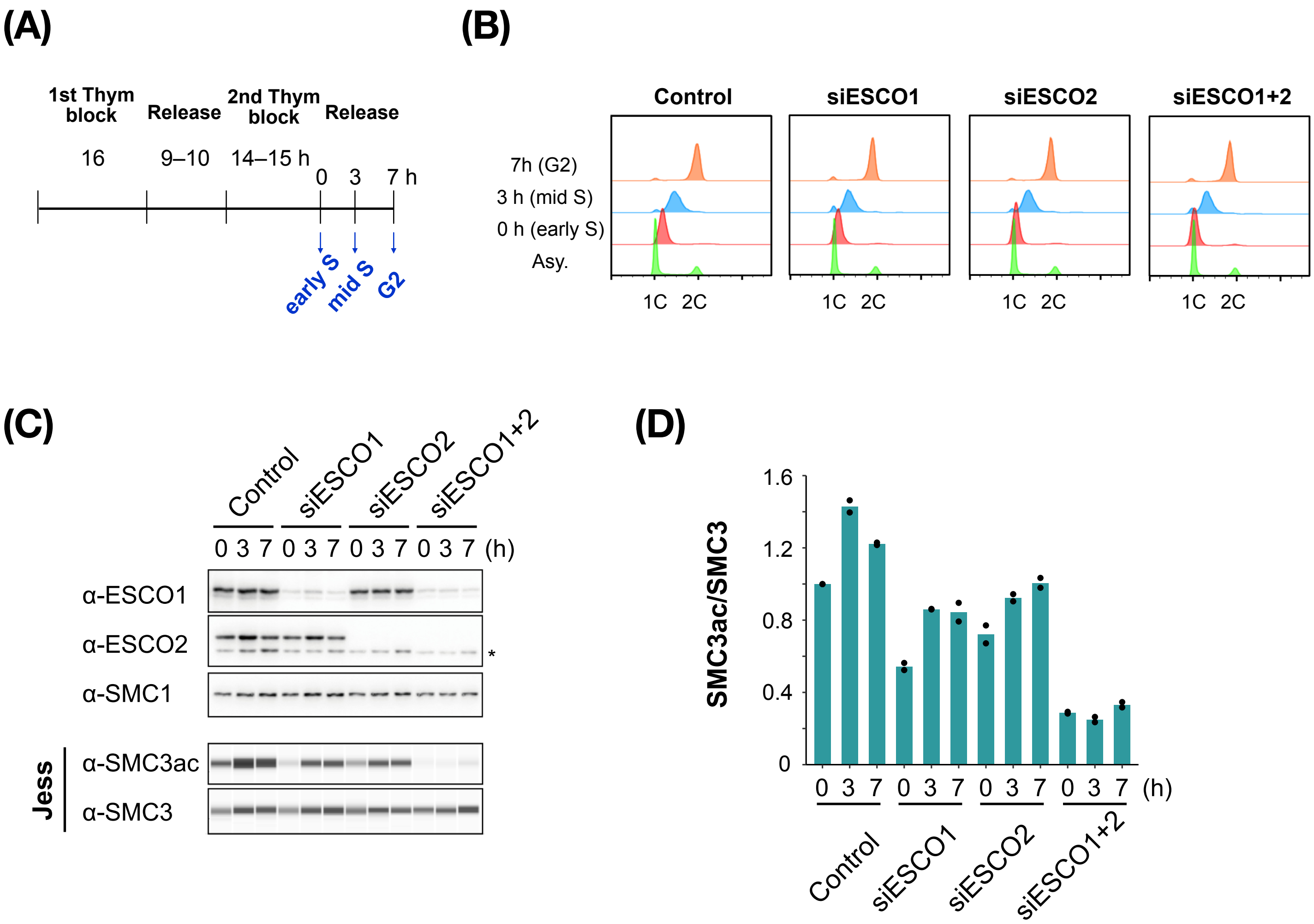

Figure S1 Cell culture validation

(A) Scheme of cell synchronization.

(B) Verification of synchronization by flow cytometry. Cells were transfected with siRNA targeting ESCO1 (siESCO1), ESCO2 (siESCO2), or both (siESCO1+2). Representative results from multiple independent experiments are shown. Asy, asynchronous.

(C) Validation of siRNA knockdown of ESCO1 and ESCO2. Protein levels of ESCO1 and ESCO2 were examined by western blotting, with the cohesin subunit SMC1 as a loading control. SMC3 acetylation levels were measured using an automated capillary western system (JESS) and presented as virtual blot images. Total SMC3 levels were also measured. Representative results from repeated experiments are shown.

(D) Ratios of SMC3ac to SMC3 measured by JESS. Results from two independent experiments are shown as dots, with the mean shown as a bar.

Figure S2

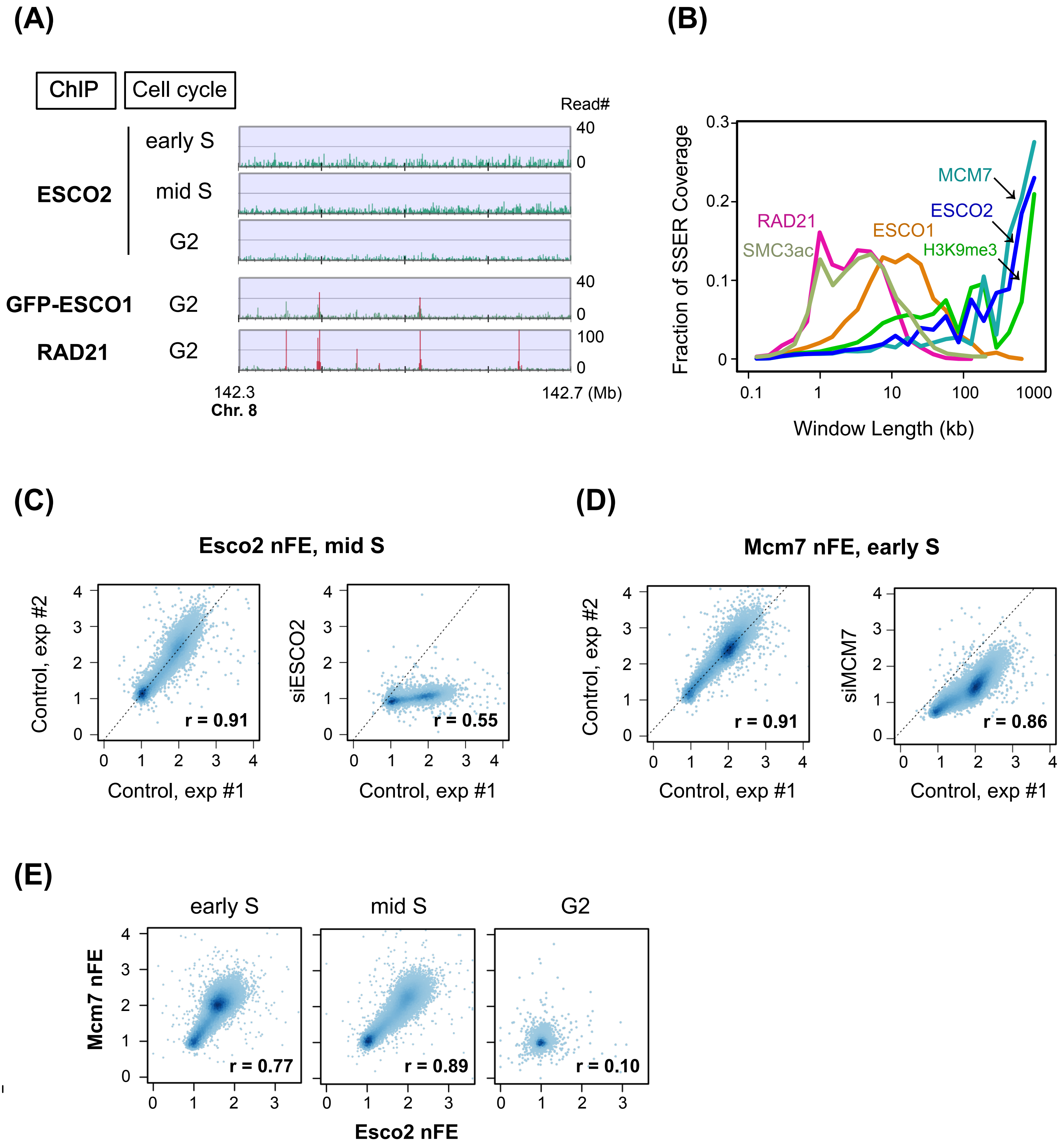
(A)
ChIP
Cell cycle
Read#
ESCO2
early S
mid S
G2
40
0
GFP-ESCO1
G2
40
0
RAD21
G2
100
0
142.3
Chr. 8
142.7 (Mb)
(B)
Fraction of SSER Coverage
0.3
0.2
0.1
0
MCM7
ESCO2
RAD21
ESCO1
SMC3ac
H3K9me3
0.1
1
10
100
1000
Window Length (kb)
(C)
Esco2 nFE, mid S
Control, exp #2
siESCO2
Control, exp #1
r = 0.91
r = 0.55
(D)
Mcm7 nFE, early S
Control, exp #2
siMCM7
Control, exp #1
r = 0.91
r = 0.86
(E)
early S
mid S
G2
Mcm7 nFE
Esco2 nFE
r = 0.77
r = 0.89
r = 0.10

Figure S2 Supplementary data for ESCO2 and MCM7 ChIP-seq analysis

(A) ChIP-seq profiles of ESCO2, GFP-ESCO1, and RAD21 at 100-bp resolution. Experimental conditions are indicated on the left. Detected peaks are highlighted in red.

(B) Distribution of enriched region sizes in ChIP-seq data for the indicated proteins, analyzed using MUSIC. SSER, scale-specific enriched region. RAD21 and SMC3ac data are from G2 cells, ESCO2 data are from mid-S cells, and MCM7 data are from early-S cells.

(C) Validation of ESCO2 ChIP-seq data. Scatter plots show high correlations of ChIP-seq nFE values between two biological replicates of untreated controls (left) and a reduction of nFE upon ESCO2 knockdown (right). Each dot represents a 100-kb bin. Data are from mid-S cells. r, Pearson's correlation coefficient.

(D) Validation of MCM7 ChIP-seq data. Scatter plots show high correlations of ChIP-seq nFE values between two biological replicates of untreated controls (left) and a reduction of nFE upon MCM7 knockdown (right). Each dot represents a 100-kb bin. Data are from early-S cells. r, Pearson's correlation coefficient.

(E) Comparison of ESCO2 and MCM7 ChIP-seq profiles across early S, mid S, and G2. ChIP-seq nFE was calculated at 100-kb resolution. r, Pearson's correlation coefficient.

Figure S3

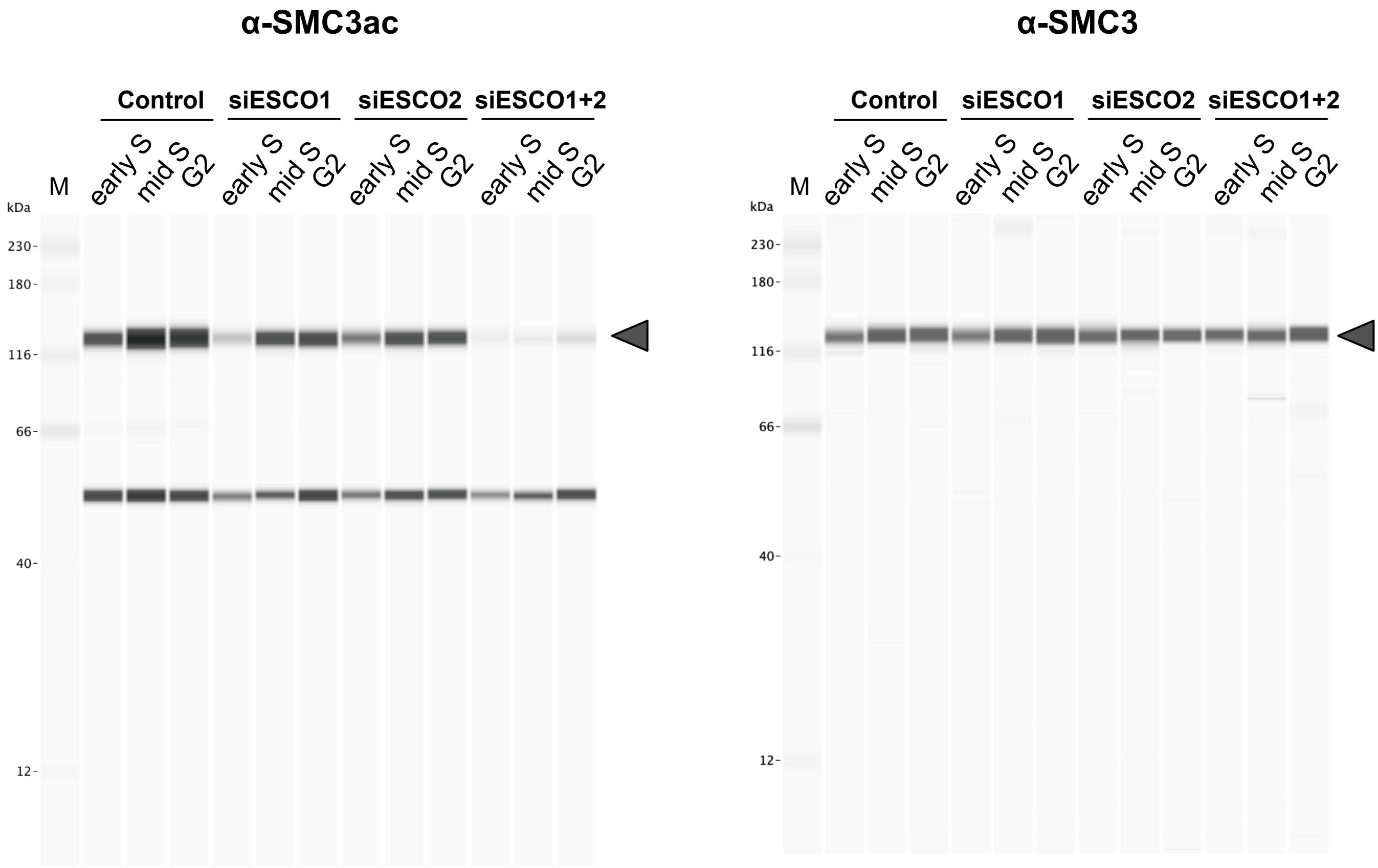

Figure S3 Antibody validation for automated capillary western system
Uncropped data corresponding to Fig. S1C. The anti-SMC3ac and anti-SMC3 antibodies detected signals at the same molecular weight (gray arrowheads), consistent with the expected size of SMC3 (~142 kDa).

Figure S4

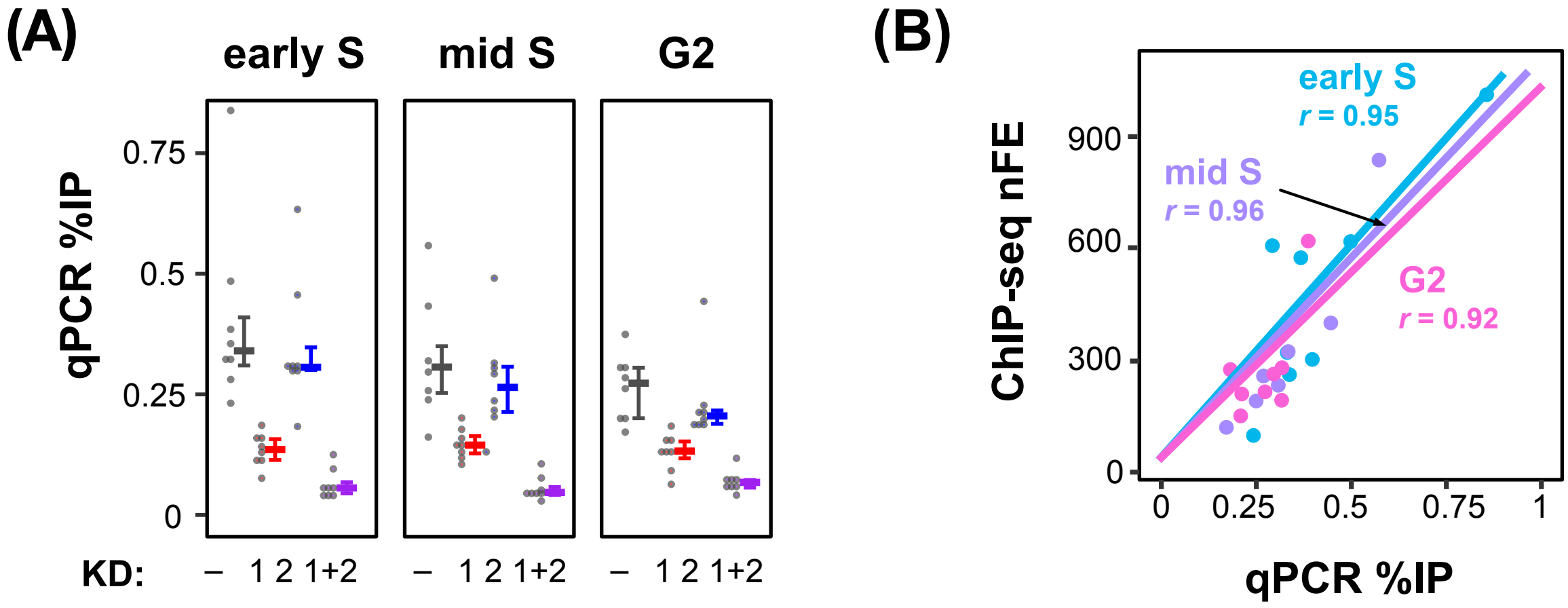

Figure S4 Validation of SMC3ac ChIP-seq data by qPCR
(A) ChIP efficiency of SMC3ac at eight cohesin peak sites quantified by qPCR. KD indicates the targeted ESCO protein(s) by siRNA. Error bars represent the interquartile range, and horizontal bold lines indicate the median.
(B) Comparison of SMC3ac ChIP efficiency measured by qPCR and ChIP-seq peak heights at the same eight cohesin peak sites in control cells without knockdown. Data from early S, mid S, and G2 cells each showed strong correlations, with similar slopes of the regression lines. These results demonstrate appropriate calibration of ChIP-seq data using spike-in controls.

Figure S5

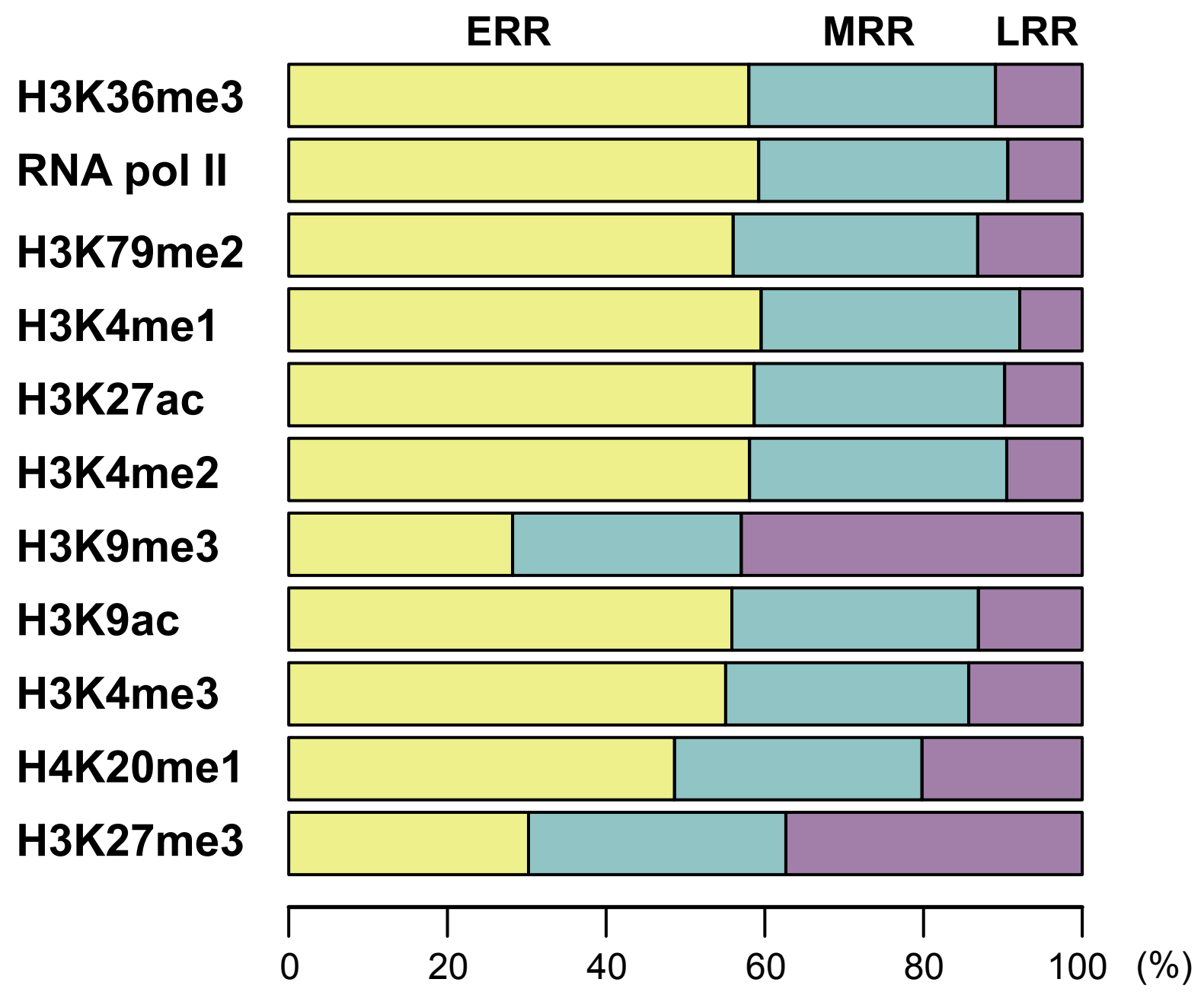

Figure S5 Replication timing distribution of histone mark-enriched genomic regions
For genomic bins with histone modification levels or RNA polymerase II binding above the median, the proportions of bins classified as ERR, MRR, and LRR are shown.

Figure S6

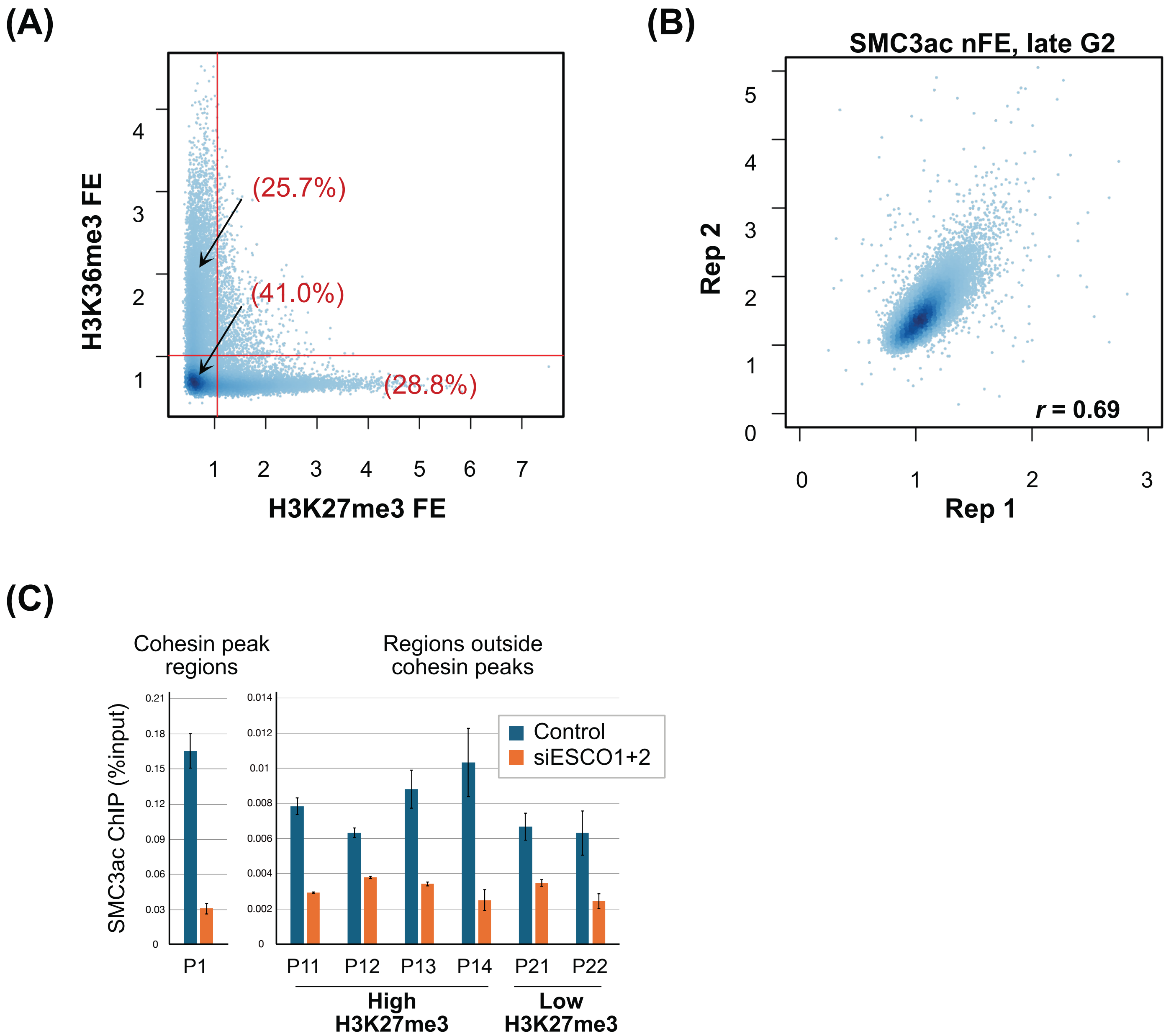

Figure S6 Supplemental data supporting preferential retention of SMC3ac in H3K27me3-marked regions

(A) Relationship between H3K27me3 and H3K36me3 levels across the genome. Each point represents a 100-kb genomic bin. Red lines indicate the genome-wide mean levels of H3K27me3 and H3K36me3. The percentages of the genome represented by regions with only H3K27me3 levels above the genome-wide mean, regions with only H3K36me3 levels above the genome-wide mean, and regions with both marks at or below their respective genome-wide means are indicated in parentheses. Together, these three categories account for 95.5% of the genome.

(B) Reproducibility of SMC3ac ChIP-seq profiles in late G2 cells. SMC3ac nFE values calculated in 100-kb bins were compared between two independent experiments. r, Pearson correlation coefficient.

(C) ChIP-qPCR analysis of chromosomal SMC3ac binding in late G2 cells. SMC3ac levels were measured in control cells and cells depleted of both ESCO1 and ESCO2 (siESCO1+2) at a cohesin peak (P1), non-peak regions with high H3K27me3 levels (P11–P14), and non-peak regions with low H3K27me3 levels (P21 and P22). Input and immunoprecipitated (IP) fractions were measured in duplicate by qPCR. Error bars indicate standard deviations calculated by error propagation.

Figure S7

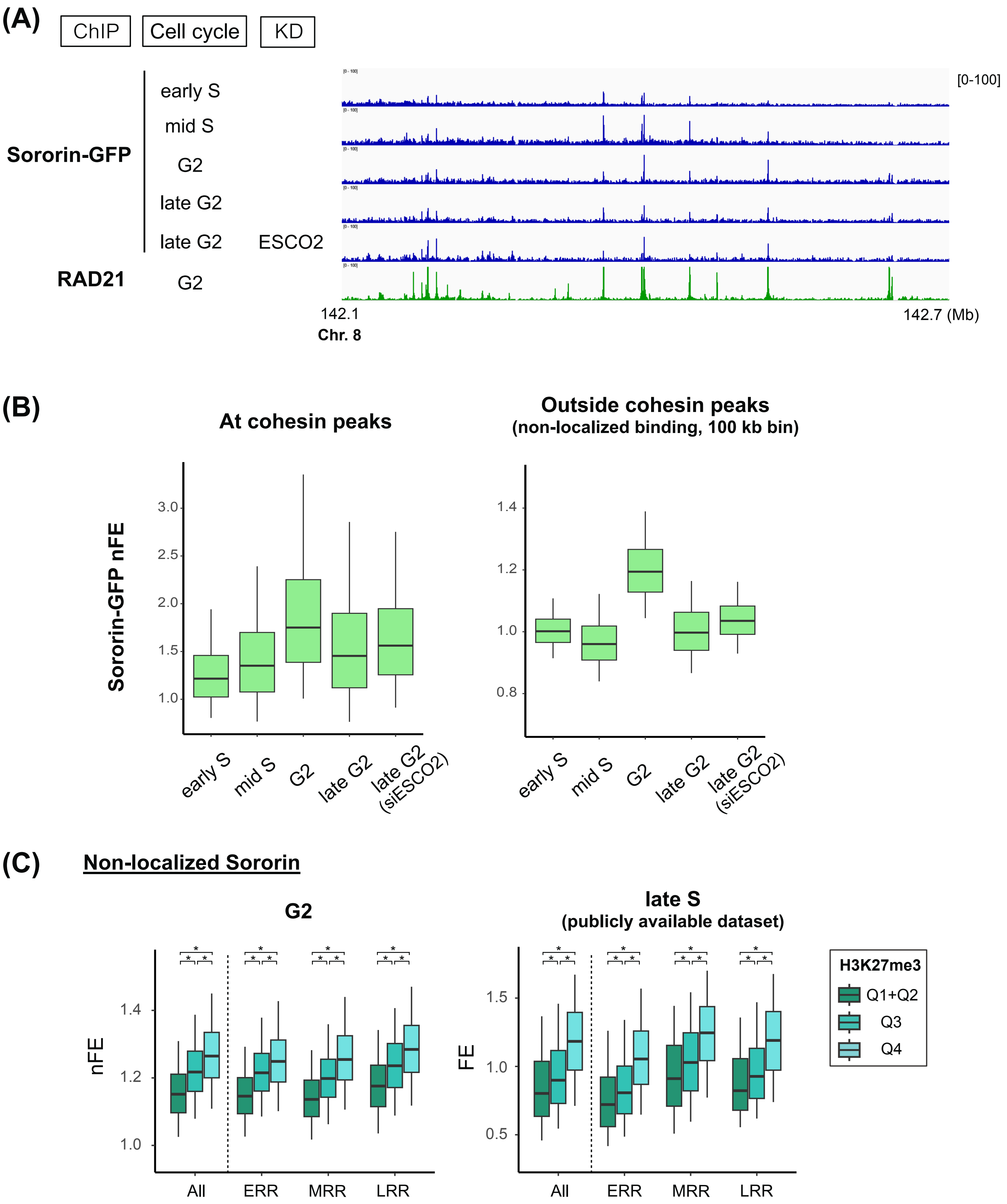
(A)
ChIP
Cell cycle
KD
Sororin-GFP
early S
mid S
G2
late G2
late G2
ESCO2
RAD21
G2
[0-100]
142.1
Chr. 8
142.7 (Mb)
(B)
At cohesin peaks
Outside cohesin peaks
(non-localized binding, 100 kb bin)
Sororin-GFP nFE
early S
mid S
G2
late G2
late G2 (siESCO2)
(C)
Non-localized Sororin
G2
late S
(publicly available dataset)
nFE
FE
All
ERR
MRR
LRR
H3K27me3
Q1+Q2
Q3
Q4

Figure S7 Analysis of Sororin by calibrated ChIP-seq

(A) Calibrated ChIP-seq profiles of Sororin-GFP. Read counts in the ChIP fractions at 100-bp resolution are shown, as in standard ChIP-seq analysis. Experimental conditions are indicated on the left. KD, knockdown by siRNA. RAD21 represents cohesin localization irrespective of its acetylation status.

(B) Quantification of chromosomal Sororin binding. ChIP-seq nFE values for cohesin peak-associated and non-localized Sororin are shown as boxplots. As for non-localized SMC3ac, non-localized Sororin nFE was calculated in 100-kb bins after excluding reads corresponding to cohesin peak regions. Boxes represent the interquartile range (25th–75th percentiles), whiskers indicate the 5th–95th percentiles, and horizontal lines within the boxes denote the median.

(C) Relationship between non-localized Sororin nFE and H3K27me3 levels. Based on H3K27me3 levels, the genome was divided into three groups (Q1+Q2: below the median, Q3: between the median and the third quartile, Q4: above the third quartile). Distributions of non-localized Sororin ChIP-seq nFE in G2 were compared across these groups. To examine whether this relationship was also observed in an independent dataset, previously reported Sororin ChIP-seq data from late S-phase cells (Ref. 41) were analyzed in the same manner (right panel). Boxes represent the interquartile range (25th–75th percentiles), whiskers indicate the 5th–95th percentiles, and bold lines denote the median. Data are shown for the whole genome (All) as well as for ERR, MRR, and LRR regions classified by replication timing. *, $P < 2.2 \times 10^{-16}$, two-sided Mann–Whitney *U* test.

Supplementary Table S1

Reagents and biological resources used in this study

REAGENT or RESOURCE	SOURCE	IDENTIFIER
Antibodies		
Mouse monoclonal anti-ESCO1	Minamino *et al*. (2015)	N/A
Rabbit polyclonal anti-RAD21	Minamino *et al*. (2015)	N/A
Mouse monoclonal anti-SMC3ac	Minamino *et al*. (2015)	N/A
Rabbit polyclonal anti-ESCO2	Abcam	Cat# ab86003; RRID:AB_1924967
Rabbit polyclonal anti-SMC1	Abcam	Cat# ab21583; RRID:AB_2192477
Rabbit monoclonal anti-SMC3 (clone D47B5)	Cell Signaling Technology	Cat# 5696, RRID:AB_10705575
Rabbit monoclonal anti-MCM7 (clone D10A11)	Cell Signaling Technology	Cat# 3735, RRID:AB_2142705
Mouse monoclonal anti-α-tubulin (clone B-5-1-2)	Sigma-Aldrich	Cat# T6074, RRID:AB_477582
Rabbit monoclonal anti-Rpb1 (clone D8L4Y)	Cell Signaling Technology	Cat# 14958, RRID:AB_2687876
Rabbit polyclonal anti-GFP	OriGene	Cat# TP401, RRID:AB_10013661
Mouse monoclonal anti-H3K27me3 (clone MABI0323)	Fujifilm	Cat# 301-95253 RRID: AB_11123929
Peroxidase-AffiniPure goat anti-Rabbit IgG	Jackson ImmunoResearch Labs	Cat# 111-035-144, RRID:AB_2307391
Peroxidase-AffiniPure goat anti-Mouse IgG	Jackson ImmunoResearch Labs	Cat# 115-035-146, RRID:AB_2307392
Chemicals, peptides, and recombinant proteins		
D-MEM (High Glucose)	Fujifilm Wako	Cat# 044-29765
Fetal Bovine Serum	Biosera	Cat# FB-1290/500
Penicillin-Streptomycin-L-Glutamine Solution	Fujifilm Wako	Cat# 161-23201
TrypLE Express Enzyme	Thermo Fisher Scientific	Cat# 12605028
Thymidine	Sigma-Aldrich	Cat# T1895-10G
RO-3306	Tocris	Cat# 4181
EPZ-6438	Selleck	Cat# S7128
Lipofectamine RNAiMAX reagent	Thermo Fisher Scientific	Cat# 13778150
cOmplete Protease Inhibitor Cocktail	Merck	Cat# 11697498001
PhosSTOP	Merck	Cat# 4906845001
Amersham Protran Premium 0.2 NC nitrocellulose Western blotting membrane	Cytiva	Cat# 10600004
Luminata Forte Western HRP substrate	Merck	Cat# WBLUF0100
Dynabeads Protein A	Thermo Fisher Scientific	Cat# DB10002
Dynabeads Protein G	Thermo Fisher Scientific	Cat# DB10004
RNase A	Merck	Cat# 10109142001

Proteinase K	Gold Biotechnology	Cat# P-480
Critical commercial assays		
QIAquick PCR Purification Kit	QIAGEN	Cat# 28106
KAPA SYBR Fast qPCR Kit	KAPA Biosystems	Cat# KK4602
NEBNext Ultra II DNA Library Prep Kit for Illumina	New England Biolabs	Cat# E7645L
Anti-Mouse Detection Module	ProteinSimple	Cat# DM-002
Anti-Rabbit NIR Detection Module	ProteinSimple	Cat# DM-007
Cell lines		
Human: HeLa S3	JCRB Cell Bank	Cat# JCRB9010
Mouse: C2C12	ECACC	Cat# 91031101
Human: HeLa expressing mouse Sororin with LAP tag	Nishiyama *et al*. (2013)	N/A
Model organisms		
Mouse: R26R-H2B-EGFP	Abe *et al*. (2011)	CDB0203K
Mouse: FVB-Tg(Ddx4-cre)1Dcas/J (Vasa-Cre)	The Jackson Laboratory	RRID:IMSR_JAX:006954
siRNA		
siRNA target sequence: ESCO1: UGAAGUAUUUGUCUUUCAACACUGG	Thermo Fisher Scientific	Assay ID:HSS132872, Cat# 1299001
siRNA target sequence: ESCO2: AUAACUUGCCAUCUGGUGUUGGGUC	Thermo Fisher Scientific	Assay ID:HSS152845, Cat# 1299001
siRNA target sequence: MCM7: CGUCACUCGUGUCUCUGAAGUCAAA	Thermo Fisher Scientific	Assay ID:HSS106405, Cat# 1299001

Supplementary Table S2

Primer pairs used for qPCR

• Coordinates are based on hg19.

• Primer sequences are shown 5′ to 3′.

Target locus ID	Chr	Start	End	Forward	Reverse
P1	chr12	53278667	53278827	TAGACGGAGCTGGAAGGAAA	AGGGACAGTCACCACCTTTG
P2	chr17	32688528	32688678	AAACCTCTGAGCTCATAATGCTG	CTGGGTGCAAGCCACACT
P3	chr1	176529142	176529282	GCAGTGTTGCTTCATTCACAGG	AAGGGATGCCAACGAACAAC
P4	chr2	54931731	54931835	TGTTGACATGTGCAGCTCAAG	TTGGATAGCTTGTTTGCTTTGC
P5	chr3	119901853	119901972	AATGCCCAGTGTCCCAAATG	TGGCGTCAGTTACCCAAAAG
P6	chr12	40501367	40501558	GCAAGGCTCTACCGTCATTC	CCTTCTCTTCAGAAGCCGTG
P7	chr15	37426978	37427078	TGTAGCAATGCTCTCTCACTCC	TGGGTCATCCACTTGGTATCAC
P8	chrX	32549072	32549144	ACCCAAAAGCTGCCTATGTC	AGCTCTGAAAGGGAATGTGC
P11	chr5	37854706	37854795	CCCCTGTCACTGTATTGTGAAG	AGCAAGACACATGGCAAAGC
P12	chr8	74029999	74030070	TGTGCGCACTCACAGGCGAA	ACAGCCCAAAGCCTTGGCACC
P13	chr6	110720591	110720670	TGGAAGAGGCGTAGGTGGCAACA	CGACCCACATGGGACGCCAC
P14	chr8	72568889	72569036	CCCCACACAACTGTCTCTGTCACCT	ACACAACTGCAGCCACCGGA
P21	chr11	94553607	94553702	TTGCTGGCAGTGCTTCATAC	TGGTGCCAAGAAGGTTGGAG
P22	chr6	38040873	38040959	ATGGGAAAGGCTCAGCAAAGAGGC	GGCCACAGGTACTGTTGCGTGGA

Supplementary Table S3
Summary of read mapping statistics

Sample description	#Total reads	#uniquely mapped reads				Occupancy Ratio	Normalization value used at the binning step	Relevant figures
		To human (hg19)		To mouse (mm10)				
earlyS_ESCO2, ChIP	27996629	23279782	83.15%	3817457	13.64%	1.2774	25547386	Fig1AC, Fig2A-D, FigS2AE
earlyS_ESCO2, Input	31481447	25223249	80.12%	5283391	16.78%	NA	20000000	Fig1AC, Fig2A-D, FigS2AE
midS_ESCO2_rep1, ChIP	38969551	33177654	85.14%	4466626	11.46%	1.2931	25862261	Fig1AC, Fig2D, FigS2A-CE
midS_ESCO2_rep1, Input	31711409	26212401	82.66%	4563281	14.39%	NA	20000000	Fig1AC, Fig2D, FigS2A-CE
G2_ESCO2, ChIP	31696139	26775804	84.48%	3879427	12.24%	0.8771	17542198	Fig1AC, Fig2D, FigS2AE
G2_ESCO2, Input	30553076	26250390	85.92%	3335914	10.92%	NA	20000000	Fig1AC, Fig2D, FigS2AE
midS_ESCO2_rep2, ChIP	36006885	30523784	84.77%	4249227	11.80%	1.5029	30058055	FigS2C
midS_ESCO2_rep2, Input	38778441	31099022	80.20%	6506526	16.78%	NA	20000000	FigS2C
midS_siESCO2_ESCO2, ChIP	36256926	28158080	77.66%	7016431	19.35%	0.8767	17533346	Fig1A, FigS2C
midS_siESCO2_ESCO2, Input	37394101	29774591	79.62%	6504199	17.39%	NA	20000000	Fig1A, FigS2C
earlyS_MCM7_rep1, ChIP	42735303	37214867	87.08%	4013755	9.39%	2.8974	57948797	Fig1AC, Fig2ABD, FigS2BDE
earlyS_MCM7_rep1, Input	45674833	33653207	73.68%	10516598	23.02%	NA	20000000	Fig1AC, Fig2ABD, FigS2BDE
midS_MCM7, ChIP	36001906	31298569	86.94%	3405543	9.46%	2.6241	52482258	Fig1AC, Fig2D, FigS2E
midS_MCM7, Input	40055798	30208377	75.42%	8625252	21.53%	NA	20000000	Fig1AC, Fig2D, FigS2E
G2_MCM7, ChIP	37824330	32565071	86.10%	4027510	10.65%	1.6381	32761318	Fig1AC, Fig2D, FigS2E
G2_MCM7, Input	36965855	29742911	80.46%	6025588	16.30%	NA	20000000	Fig1AC, Fig2D, FigS2E
earlyS_MCM7_rep2, ChIP	44295023	39446907	89.05%	3303192	7.46%	3.6745	73489234	FigS2D
earlyS_MCM7_rep2, Input	43454938	32152642	73.99%	9893074	22.77%	NA	20000000	FigS2D
earlyS_siMCM7_MCM7, ChIP	46967096	39932928	85.02%	5368054	11.43%	2.1367	42734314	Fig1A, FigS2D
earlyS_siMCM7_MCM7, Input	45323216	34002754	75.02%	9766671	21.55%	NA	20000000	Fig1A, FigS2D
earlyS_RAD21, ChIP	37558919	30452910	81.08%	6071724	16.17%	1.5653	31306104	Fig4C
earlyS_SMC3ac, ChIP	41366732	33323096	80.56%	6383352	15.43%	1.6292	32584330	Fig3A-C, Fig4A, Fig5A, Fig6B, FigS4B
earlyS_RAD21/SMC3ac, Input	35919675	26600446	74.06%	8301779	23.11%	NA	20000000	Fig1B, Fig3A-C, Fig4A, Fig5A, Fig6B, FigS4B
earlyS_siESCO1_SMC3ac, ChIP	40627452	31123950	76.61%	7792496	19.18%	1.2132	24263210	Fig3C, Fig4A, Fig5A
earlyS_siESCO1_SMC3ac, Input	38290301	28536989	74.53%	8667788	22.64%	NA	20000000	Fig3C, Fig4A, Fig5A
earlyS_siESCO2_SMC3ac, ChIP	40088867	30770312	76.76%	7557174	18.85%	1.3164	26327926	Fig3C, Fig4A, Fig5A
earlyS_siESCO2_SMC3ac, Input	36797528	27003723	73.38%	8730474	23.73%	NA	20000000	Fig3C, Fig4A, Fig5A
earlyS_siESCO1&2_SMC3ac, ChIP	40259257	27476464	68.25%	10741597	26.68%	0.8280	16560502	Fig3C, Fig4A, Fig5A
earlyS_siESCO1&2_SMC3ac, Input	38060254	27930385	73.38%	9041249	23.76%	NA	20000000	Fig3C, Fig4A, Fig5A
midS_RAD21, ChIP	38183615	30681683	80.35%	6430558	16.84%	1.2496	24991178	Fig4C
midS_SMC3ac, ChIP	38197108	32302958	84.57%	4631611	12.13%	1.8266	36531410	Fig3A-C, Fig4A, Fig5A, Fig6B, FigS4B
midS_RAD21/SMC3ac, Input	35133512	27095054	77.12%	7096043	20.20%	NA	20000000	Fig1B, Fig3A-C, Fig4A, Fig5A, Fig6B, FigS4B
midS_siESCO1_SMC3ac, ChIP	40278194	32734342	81.27%	6253823	15.53%	1.3454	26907125	Fig3AC, Fig4A, Fig5A
midS_siESCO1_SMC3ac, Input	38476897	29788846	77.42%	7656547	19.90%	NA	20000000	Fig3AC, Fig4A, Fig5A
midS_siESCO2_SMC3ac, ChIP	39916094	31964012	80.08%	6495771	16.27%	1.3343	26686149	Fig3AC, Fig4A, Fig5A
midS_siESCO2_SMC3ac, Input	36118713	27641998	76.53%	7495399	20.75%	NA	20000000	Fig3AC, Fig4A, Fig5A
midS_siESCO1&2_SMC3ac, ChIP	43998490	30796290	69.99%	10452142	23.76%	0.8659	17317016	Fig3AC, Fig4A, Fig5A
midS_siESCO1&2_SMC3ac, Input	36856543	27718462	75.21%	8145525	22.10%	NA	20000000	Fig3AC, Fig4A, Fig5A
G2_RAD21, ChIP	39002938	33266067	85.29%	4569576	11.72%	1.3517	27033226	Fig3AB, Fig4C, FigS2AB
G2_SMC3ac, ChIP	40540435	34757866	85.74%	4338511	10.70%	1.4875	29749846	Fig3A-C, Fig4AB, Fig5A, Fig6AB, FigS2B, FigS4B
G2_RAD21/SMC3ac, Input	38714248	31694280	81.87%	5884685	15.20%	NA	20000000	Fig1B, Fig3A-C, Fig4A-C, Fig5A, Fig6AB, FigS2AB, FigS4B
G2_siESCO1_SMC3ac, ChIP	40993679	34331438	83.75%	4824845	11.77%	1.4154	28307011	Fig3C, Fig4AB, Fig5A
G2_siESCO1_SMC3ac, Input	36494541	29546652	80.96%	5877108	16.10%	NA	20000000	Fig3C, Fig4AB, Fig5A
G2_siESCO2_SMC3ac, ChIP	39913920	32753298	82.06%	5524340	13.84%	1.2585	25170654	Fig3C, Fig4AB, Fig5A
G2_siESCO2_SMC3ac, Input	41022955	32870452	80.13%	6977431	17.01%	NA	20000000	Fig3C, Fig4AB, Fig5A
G2_siESCO1&2_SMC3ac, ChIP	40109527	31699433	79.03%	6999559	17.45%	0.9624	19247294	Fig3C, Fig4AB, Fig5A
G2_siESCO1&2_SMC3ac, Input	38903183	31149943	80.07%	6619362	17.01%	NA	20000000	Fig3C, Fig4AB, Fig5A
lateG2_SMC3ac_rep1, ChIP	40195549	33341356	82.95%	5386531	13.40%	0.8698	17396143	Fig5A, Fig6A, FigS6B
lateG2_SMC3ac_rep1, Input	35768332	30383357	84.94%	4269575	11.94%	NA	20000000	Fig1B, Fig5A, Fig6A, FigS6B
G2_siRAD21_RAD21, ChIP	41740686	15140785	36.27%	25388196	60.82%	0.2121	4241055	Fig3B
G2_siRAD21_SMC3ac, ChIP	35621020	22055338	61.92%	12236141	34.35%	0.6409	12818188	Fig3B
G2_siRAD21_SMC3ac, Input	40846457	31771424	77.78%	7900311	19.34%	NA	20000000	Fig3B
Asy_Rpb1 (RNA pol II), ChIP	37482563	30448540	81.23%	NA	NA	NA	20000000	Fig2AC, Fig6A
Asy_Rpb1 (RNA pol II), Input	55793132	42466802	76.11%	NA	NA	NA	20000000	Fig2AC, Fig6A
lateG2_SMC3ac_rep2, ChIP	74888861	60481288	80.76%	7514676	10.03%	1.8538	37076462	Fig6BC, FigS6B
lateG2_H3K27me3, ChIP	74392457	62399938	83.88%	3458553	4.65%	4.1557	83113875	Fig6C
lateG2_SMC3ac(rep2)/H3K27me3, input	81591847	59663855	73.12%	13742423	16.84%	NA	20000000	Fig6BC, FigS6B
lateG2_EZP6438_SMC3ac_rep2, ChIP	71,808,565	57,815,683	80.51%	7,301,420	10.17%	1.3981	27962569	Fig6C
lateG2_EZP6438_H3K27me3, ChIP	81,203,392	55,816,609	68.74%	18,044,835	22.22%	0.5462	10923410	Fig6C
lateG2_EZP6438_SMC3ac(rep2)/H3K27me3, input	70,432,024	53,799,358	76.38%	9,499,080	13.49%	NA	20000000	Fig6C
earlyS_Sororin-GFP, ChIP	257208002	107289812	41.71%	140044597	54.45%	0.1962	3923736	FigS7A-C
earlyS_Sororin-GFP, input	65902510	48145718	73.06%	12330138	18.71%	NA	20000000	FigS7A-C
midS_Sororin-GFP, ChIP	75417894	33438811	44.34%	38829101	51.49%	0.2073	4146310	FigS7A-C
midS_Sororin-GFP, input	88458437	65141024	73.64%	15682387	17.73%	NA	20000000	FigS7A-C
G2_Sororin-GFP, ChIP	77564486	42001076	54.15%	30403831	39.20%	0.237	4739944	FigS7A-C
G2_Sororin-GFP, input	73833738	56769441	76.89%	9739014	13.19%	NA	20000000	FigS7A-C
lateG2_Sororin-GFP, ChIP	62910605	29620140	47.08%	30350384	48.24%	0.1972	3943158	FigS7A-C
lateG2_Sororin-GFP, input	76196817	57444414	75.39%	11604561	15.23%	NA	20000000	FigS7A-C
lateG2_siESCO2_Sororin-GFP, ChIP	81691563	37090626	45.40%	41069491	50.27%	0.2102	4204611	FigS7A-C
lateG2_siESCO2_Sororin-GFP, input	130870222	96312850	73.59%	22421014	17.13%	NA	20000000	FigS7A-C

Supplementary Table S4

Publicly available sequencing data used in this study

All datasets were derived from HeLa S3 cells.

Description	File ID/ Accession ID	Download site
GFP-ESCO1, ChIP-seq in G2	SRR1916173	https://trace.ncbi.nlm.nih.gov/Traces?run=SRR1916173
Replication timing by Repli-seq (wavelet-smoothed signal)	GSM923449_hg19_wgEncodeUwRepliSeqHelas3WaveSignalRep1.bigWig	https://www.ncbi.nlm.nih.gov/geo/download/?acc=GSM923449&format=file&file=GSM923449%5Fhg19%5FwgEncodeUwRepliSeqHelas3WaveSignalRep1%2EbigWig
H3K4me1, ChIP-seq	E117-H3K4me1.tagAlign.gz	https://egg2.wustl.edu/roadmap/data/byFileType/alignments/consolidated/
H3K4me2, ChIP-seq	E117-H3K4me2.tagAlign.gz	
H3K4me3, ChIP-seq	E117-H3K4me3.tagAlign.gz	
H3K9ac, ChIP-seq	E117-H3K9ac.tagAlign.gz	
H3K27ac, ChIP-seq	E117-H3K27ac.tagAlign.gz	
H3K27me3, ChIP-seq	E117-H3K27me3.tagAlign.gz	
H3K36me3, ChIP-seq	E117-H3K36me3.tagAlign.gz	
H3K79me2, ChIP-seq	E117-H3K79me2.tagAlign.gz	
H4K20me1, ChIP-seq	E117-H4K20me1.tagAlign.gz	
Input for ChIP (roadmap)	E117-Input.tagAlign.gz	
H3K9me3, ChIP-seq (Fold change over control)	ENCFF761QZP	https://www.encodeproject.org/files/ENCFF761QZP/
Sororin, ChIP-seq in late S	ERR1194877	https://www.ebi.ac.uk/ena/browser/view/ERR1194877
Genome control for Sororin ChIP	ERR1195873	https://www.ebi.ac.uk/ena/browser/view/ERR1195873
RNA-seq	SRR16555324	https://trace.ncbi.nlm.nih.gov/Traces/?view=run_browser&acc=SRR16555324&display=download
